# A profile of the online dissemination of national influenza surveillance data

**DOI:** 10.1186/1471-2458-9-339

**Published:** 2009-09-16

**Authors:** Calvin KY Cheng, Eric HY Lau, Dennis KM Ip, Alfred SY Yeung, Lai Ming Ho, Benjamin J Cowling

**Affiliations:** 1 Department of Community Medicine and School of Public Health, the University of Hong Kong, Hong Kong

## Abstract

**Background:**

Influenza surveillance systems provide important and timely information to health service providers on trends in the circulation of influenza virus and other upper respiratory tract infections. Online dissemination of surveillance data is useful for risk communication to health care professionals, the media and the general public. We reviewed national influenza surveillance websites from around the world to describe the main features of surveillance data dissemination.

**Methods:**

We searched for national influenza surveillance websites for every country and reviewed the resulting sites where available during the period from November 2008 through February 2009. Literature about influenza surveillance was searched at MEDLINE for relevant hyperlinks to related websites. Non-English websites were translated into English using human translators or Google language tools.

**Results:**

A total of 70 national influenza surveillance websites were identified. The percentage of developing countries with surveillance websites was lower than that of developed countries (22% versus 57% respectively). Most of the websites (74%) were in English or provided an English version. The most common surveillance methods included influenza-like illness consultation rates in primary care settings (89%) and laboratory surveillance (44%). Most websites (70%) provided data within a static report format and 66% of the websites provided data with at least weekly resolution.

**Conclusion:**

Appropriate dissemination of surveillance data is important to maximize the utility of collected data. There may be room for improvement in the style and content of the dissemination of influenza data to health care professionals and the general public.

## Background

Upper respiratory viruses cause significant global mortality and morbidity each year [1]. Influenza virus is of particular public health concern due to its association with severe infections and deaths, and its propensity of causing large scale seasonal epidemics and pandemics. Local and national prospective influenza and influenza-like illness surveillance systems provide important and timely information to policy makers and public health practitioners for monitoring trends and disease burden, planning, implementing, and evaluating appropriate prevention and control interventions, and allocating resources [2]. Recent decades have seen dramatic improvements in influenza surveillance systems [3]. Surveillance websites serve as an excellent tool for communicating timely information about disease activity to health care professionals, the media and the general public.

**Figure 1:**
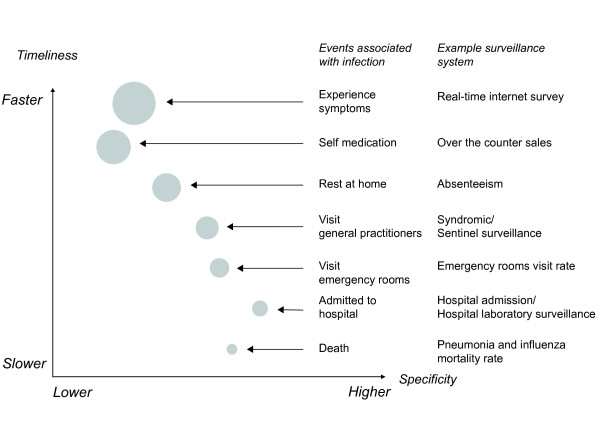
**Schematic diagram of the course of illness and clinical iceberg of upper respiratory infections in a population, and examples of surveillance systems targeting each stage**. The exact proportion of infections falling into each category, and the specificity of surveillance data to influenza will vary at different times and in different settings.

There are several surveillance methods to track influenza virus activity. Each method only captures a portion of infections within the community with different timeliness and specificity (Figure 1). Laboratory surveillance of viral culture or molecular methods on specimens taken during acute infections can provide highly specific information on influenza virus activity [4], but test results can often take at least one to two weeks. Also this tends to capture cases with more severe infections including hospitalized patients, while people with milder infections may not seek medical care and thus would not contribute specimens to the surveillance system. Syndromic surveillance of influenza-like illness at emergency rooms and outpatient clinics may provide more timely data although not as specific to influenza virus activity [5-7]. Surveillance of school and workplace absenteeism, or over-the-counter pharmaceuticals usage may capture a larger proportion of influenza virus infections, but may be affected by the activity of other upper respiratory pathogens.

In this systematic review, we summarized the websites of national influenza and influenza-like illness surveillance systems from around the world. We studied the features of these websites and their underlying databases. This review may serve as a reference for future comparison and to evaluate improvements in the dissemination of influenza surveillance data.

## Methods

### Study Timing

The study was conducted during the period between November 1, 2008 to February 3, 2009. All online searches were carried out from November 1 to December 14, 2008 and website features retrieval were accessed for three times within the period December 15, 2008 to February 3, 2009 (first attempt: December 15-30, 2008; second attempt: January 19-22, 2009; third attempt: February 2-3, 2009).

### Inclusion and exclusion criteria

We aimed to include all websites in any language describing national prospective influenza and/or influenza-like illness surveillance systems. Surveillance websites that only describe surveillance data on avian influenza were not included. We also excluded websites with apparent delays of more than one year in providing influenza surveillance data. For those countries which had more than one website, we selected the official national website for analysis.

### Search strategy

#### Review of international influenza surveillance networks

Currently (up to February 3, 2009) the World Health Organization (WHO) has the largest human seasonal influenza surveillance network in the world, consisting of 122 institutions from 94 countries which monitor the circulating strains and decide the content of the influenza vaccine for the next influenza season [8]. We explored this website for relevant information and hyperlinks. Other well known influenza networks such as the Centers for Disease Control and Prevention (CDC) of the United States [9], the European Influenza Surveillance Scheme (EISS) [10], Pandemicflu.ca [11], FluWiki website [12] and the Network for Communicable Disease Control in Southern Europe and Mediterranean Countries [13] were also explored for relevant information and hyperlinks. For countries that had no hyperlink or no information for their national health agency in the above websites, we conducted Google searches using the query (("Ministry of Health" OR "Department of Health") AND "<country name>") to locate the corresponding influenza surveillance websites. We investigated websites from the top 50 hits for each search result.

#### Screening of existing literature

Literature about influenza surveillance was extensively searched at MEDLINE for relevant articles published in all years using the following search strategy:

 #1 "influenza" [All Fields]

 #2 "epidemiology" [All Fields] OR "surveillance" [All Fields]

 #3 "online" [All Fields] OR "website" [All Fields]

 #1 AND #2 AND #3

Useful information about surveillance data dissemination and any relevant internet hyperlinks were extracted for further analysis. For literature without hyperlinks, we tried to contact the corresponding authors for relevant information.

#### Search of existing national influenza surveillance websites

Influenza surveillance related websites were searched in every country listed in Wikipedia , 216 countries in total, excluding countries in Antarctica using the Google search engine with keywords "influenza surveillance" and the specific English name of the country, i.e. the search strategy: ("<country name>" AND "influenza surveillance" AND ("website" OR "online")). For each search we extracted relevant information from the top 50 hit websites. Queries were translated by Google language tools  to local official languages and searches were repeated to further increase our scope. We searched for websites in languages including Arabic, Chinese, French, German, Italian, Japanese, Korean, Portuguese and Spanish.

### Data Extraction

Information was extracted either directly from the literature or by personal communication. Two authors (CKYC and ASYY) independently extracted characteristics of the surveillance websites in terms of data quality and dissemination, which included type, resolution, timeliness and coverage of surveillance data, as well as presentation, ease of data retrieval and interpretation of the data. Websites with discrepant characteristics were reviewed again and consensus was made through detailed discussion between the two authors. When consensus could not be reached, all authors were consulted before making a final decision. For non-English websites, Google language tools were used to translate web pages into English to extract relevant information for analysis.

## Results

The MEDLINE search yielded 28 articles related to influenza surveillance. We identified 5 influenza surveillance websites based on the content of these reviews [14-18]. We identified 33 influenza surveillance websites by exploring those influenza surveillance network websites. 94 relevant websites were found by Google. In total we found 132 websites in 98 countries and we retained 70 websites based on our selection criteria for further analysis.

**Figure 2:**
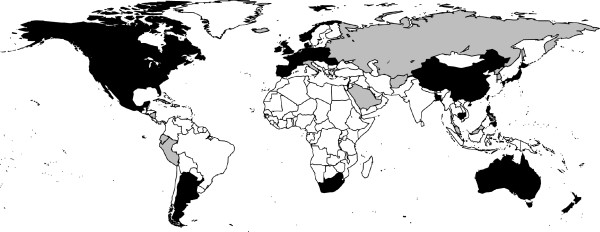
**Online influenza surveillance websites worldwide**. Nations with influenza or influenza-like illness surveillance websites included in this review are shaded gray. Nations with laboratory influenza surveillance data are shaded black.

Figure 2 shows the geographical location of those countries which had influenza surveillance data available. The percentage of developing countries with surveillance websites was lower than that of developed countries (33/151, 22% versus 37/65, 57% respectively); 38/70 (54%) were official national websites and 52/70 (74%) websites were either in English or provided an English version. Hyperlinks to these websites are provided in the online appendix [19].

### Type of surveillance data

The most common surveillance data included community influenza-like illness rates collected by general practitioners (62/70, 89%) and laboratory surveillance of acute infections confirmed either by viral culture, RT-PCR or Hemagglutination Inhibition (HI) test (31/70, 44%). Influenza sub-typing for influenza A viruses (either H1, H3 or H5) were available in 81% of those websites that contained laboratory data. Other types of surveillance data, such as antiviral usage or antiviral resistance, were rarely reported.

Other surveillance data included community institutional outbreaks, point-of-care rapid influenza tests, pneumonia and influenza mortality, hospital admissions and emergency room visits. School or work place absenteeism and emergency phone calls due to influenza-like illness were less common (Table 1). More than half of the websites (37/70, 53%) only reported one type of data for influenza activity surveillance, while 15 websites have 3 or more indicators to reflect influenza activity in different sectors of the community. Only about one third (26/70, 37%) of the surveillance websites provided a clear definition of the case definition (numerator) and the source population (denominator).

**Table 1:** Characteristics of influenza surveillance systems used in different countries

**Surveillance Methods**	**Number (%) **	**of countries (n = 70)**
Community consultation rates of influenza-like illness	62	(89%)
Virological data by viral culture/RT-PCR and/or HI assays	31	(44%)
Number of institutional Outbreaks	9	(13%)
Hospital admission rates	7	(10%)
Mortality (Pneumonia and influenza) rates	7	(10%)
Emergency room visits due to influenza-like illness	5	(7%)
School/Workplace absenteeism rates	5	(7%)
Real-time internet survey	4	(6%)
Antiviral resistant strain surveillance	4	(6%)
Quick immunological assays data	1	(1%)
Over the counter sales	1	(1%)
Others	7	(10%)

### Resolution, timeliness and coverage of surveillance data

Except for 3 websites which provide real time self-reported influenza-like illness symptoms data via the internet, no websites have data with a daily resolution. More than half of the websites (46/70, 66%) provided surveillance data with weekly resolution, while others disseminated the data monthly to yearly, particularly in those developing countries. Only 14 websites (14/70, 20%) provided age-specific surveillance data, and even less websites (2/70, 3%) provided sex-specific data. Several websites (27/70, 39%) provided data stratified by geographical regions within the countries. Some incorporated a graphical geographical information system (GIS) which presented data on geographic maps with colour code to represent levels of influenza activity. Regarding timeliness, on average the reports had a reporting delay of around 2 weeks for syndromic surveillance, and longer for reports of laboratory data. While most websites provided information about the source of the surveillance data in their websites, not many of them (15/70, 21%) provided information on surveillance coverage (eg. average sentinel points per kilometer).

### Data presentation

Common data presentation features of current existing influenza surveillance reports are summarized in Table 2. Most websites (49/70, 70%) presented data in a report format. In many cases, a summary paragraph was found on the first page of the report followed by surveillance data in tables and/or graphs (55/70, 79%) including comparisons with historical data. The median length of surveillance data shown in time series graphs, if provided, was 1 year (range: 3 months - 9 years). Special highlights (eg. sudden rise of influenza activity within a short period of time) were generally stated in the summary paragraph, whereas for those report data in graphs, 19% also indicated highlights directly on their graphical presentations using special symbols and/or colors.

**Table 2:** Data presentation features of national influenza surveillance websites

**Features of data presentation**	**Number (%) of websites found (n = 70)**
Summary paragraph	56 (80%)
Graphical and/or tabular display	55 (79%)
Data in weekly resolution	46 (66%)
More than one year time series data presented	37 (53%)
Geographical of influenza activity	27 (39%)
Source of data provided	26 (37%)
Coverage of data provided	15 (21%)
Age specific data provided	14 (20%)
Raw data export	3 (4%)
Interactive data retrieval	2 (3%)
Sex specific data provided	2 (3%)

### Data retrieval

Only 3/70 (4%) websites provided raw data export, in standard data formats (eg. Comma Separated Values) for further usage. Dynamic data retrieval functions are available in 2 of these 3 websites for end users (The SurvStat@RKI system from Germany [20] and the SmiNet-2 system from Sweden [21]) These websites provided a platform for interactive data search and retrieval that allowed users to extract data according to their specific needs.

### Data interpretation

Data interpretation of these reports was often limited to empirical summaries. Nearly all websites only gave descriptive statistics of the data with little interpretation, while some (8/70, 11%) interpreted the data briefly as overall situation in terms of a 5 point scale, i.e. from no activity, sporadic, local, regional and widespread activity. Only a few websites (4/70, 6%) included a calculated threshold for defining influenza activity levels. Very few websites (6/70, 9%) provided advice for the general public specific to the current influenza activity level. No websites attempted to quantitatively forecast future influenza activity using mathematical modeling or other algorithms.

## Discussion

The primary aim of infectious disease surveillance is to provide useful information that can be utilized by different relevant parties, including health care experts as well as the general public for appropriate disease control measures. The success of a disease surveillance system requires good quality data disseminated timely and efficiently, which depends on several factors such as surveillance coverage, data accuracy, timeliness of data acquisition and dissemination, and effectiveness of data presentation [2]. Publicly available disease surveillance websites provide an excellent channel for timely sharing disease surveillance data and risk communication. In this article we profiled websites of national influenza surveillance systems.

Not many websites provide surveillance data stratified by age or location which may unmask partly the transmission dynamics in terms of spatial distribution or population mixing. Besides, although historical surveillance data were available in some websites' archives, many reports only provided influenza activity data for the current year. For prospective disease surveillance it could be useful to interpret current data in comparison to reference data from previous years.

Surveillance data dissemination in weekly resolution would delay the public health responses when there is a sudden change of disease activities, especially in the rising phase and during the evolving phase of an epidemic or pandemic situation. Newer approaches such as quick immunological assays and school/work absenteeism surveillance can be considered for more timely data dissemination, whereas combination analysis of these data may generate higher specificity for influenza activities.

An ideal surveillance website should have comparable hardware and software, standard user interface, data format and coding for easy data sharing [2]. However, presentation of data in the websites studied were often static and in different formats, making it difficult even to copy and paste out of the website. When surveillance data in websites are generated from back-end databases it should be straightforward to include raw data export functions for standard formats such as Comma Separated Values (CSV) or Extensible Markup Language (XML) format.

A primary motivation for publicizing surveillance data online should be to allow timely risk communication thereby facilitating disease prevention [22], however most of the websites only described the surveillance data and the levels of influenza activity without providing advice on appropriate actions. Supported by surveillance data, health agencies could take the opportunity to publicize in their websites the public health interventions which could be taken by public health practitioners, organizations (e.g. schools) or the general public [2,23].

This study was carried out in early 2009 prior to the emergence of pandemic influenza A (H1N1). This review may serve as a baseline study for future comparisons of improvements and updates in influenza surveillance data sharing and dissemination following the pandemic.

### Limitations

This review has several limitations. Firstly, only public websites and/or databases were included for analysis. We were not able to access intranets which might provide more detailed data to registered users. Some non-English websites may have been missed in our searching procedures and we intend to maintain and continue to update the online appendix with hyperlinks to national influenza surveillance websites. This review only investigated human influenza surveillance, and avian influenza surveillance websites were excluded. However, we emphasize effective data sharing and risk communication at local as well as international level in this review, our analysis should not be affected by those unreachable websites as only those publicly available and easily located surveillance websites will be most effective to serve these purposes. Though our review was mainly based on quantitative measures, some of the assessment may still involve subjective interpretation. We minimized subjectivity by establishing clear definitions before data extraction, evaluating each websites by at least two authors. More subjective characteristics such as design and readability were not directly reviewed but were assessed through other quantitative measures such as usage of tables, graphs and highlights. Finally, our review focuses on the dissemination and interpretation of surveillance data, and has not reviewed the underlying surveillance systems [2].

## Conclusion

While the quality of influenza surveillance data can be improved by investing more resources, less attention has been given to the dissemination of surveillance data and the translation of information into action. Advances in infectious disease informatics research in recent years has allowed significant improvements in data collection, sharing, reporting, analyzing, and data visualization, which allows better data presentation and interpretation on surveillance websites for maximizing the data usage.

The comments and recommendations above may be generalizable to other infectious diseases. Surveillance data therefore can be fully utilized by stakeholders in a convenient and timely way for more efficient resource allocation as well as strategic planning for infectious disease control.

## Competing interests

The authors declare that they have no competing interests.

## Authors' contributions

CYKC participated in data collection, and interpreting the results. EHYL participated in planning the study, data collection, and interpreting the results. DKMI participated in interpreting the results. ASYY participated in data collection, and interpreting the results. LMH participated in interpreting the results. BJC participated in planning the study, data collection, and interpreting the results. EHYL and BJC obtained research funding. CKYC wrote the first draft of the article. All authors were involved in planning the article, critical review and editing of the first draft, and subsequent revisions to the paper. All authors read and approved the final manuscript.

## Pre-publication history

The pre-publication history for this paper can be accessed here:


